# Insights into the Nanobiology of Growth Hormone Secretion

**DOI:** 10.15190/d.2014.14

**Published:** 2014-09-15

**Authors:** Lloyd L. Anderson

**Affiliations:** Department of Animal Science, College of Agriculture and Life Sciences and Department of Biomedical Sciences, College of Veterinary Medicine, Iowa State University, Ames, IA 50011-3150 USA

**Keywords:** GH cells, Secretory vesicles, Exocytosis

## Abstract

The fact that partially empty vesicles are generated following cell secretion suggested that secretory vesicles do not collapse at the cell plasma membrane but, rather, transiently dock and fuse at the plasma membrane to expel a portion of their contents before retracting or undergoing endocytosis into the cell. Such a process has also been referred to in the literature as a “kiss-and-run” mechanism. This mechanism of cell secretion was conclusively demonstrated following the discovery of permanent cup-shaped lipoprotein structures at the cell plasma membrane, called “porosomes”, where secretory vesicles transiently dock and fuse to expel intravesicular contents from the cell. Porosomes are present in all secretory cells, from the digestive enzyme-secreting pancreatic acinar cells, to the hormone-releasing growth hormone cells, mast cells, chromaffin cells, hair cells of the inner ear, to neurons secreting neurotransmitters. Hence, it can be asserted that porosomes are the universal secretory machinery in the plasma membrane of secretory cells. Therefore, the discovery of the porosome has resulted in a paradigm shift in our understanding of cell secretion. Rapid transport of secretory vesicles containing hormones to the plasma membrane is powered by high-energy molecules such as ATP, GTP or NADH. Immunogold labeled transmission electron microscopy (TEM) was used to determine the total number of secretory vesicles in resting and in GH-stimulated porcine pituitary cells. We identified three categories of vesicles: filled, empty, and partly empty. Resting GH cells contained more than twice as many filled vesicles than did the stimulated ones. However, stimulated cells contained nearly twice as many empty vesicles and 2.5 times more partly empty vesicles than did resting cells. Secretory vesicles in GH cells further revealed the localization of GH only in electron dense vesicles in both resting and stimulated cells. No change in the total number of secretory vesicles following secretion was observed. These results are consistent with a mechanism that, after stimulation of secretion, vesicles transiently dock and fuse at the porosome to establish a fusion pore, through which intravesicular contents are released.

## INTRODUCTION

It is commonly accepted that exocytosis requires incorporation of secretory vesicle membrane into the cell plasma membrane for expulsion of vesicular contents; however, studies in the last decade^1-9^ demonstrate otherwise. Physiological processes such as neurotransmission, and the secretion of enzymes and hormones, require fusion of membrane-bounded secretory vesicles at the cell plasma membrane and rapid expulsion of vesicular contents. Earlier transmission electron microscopy (TEM) studies on mast cells demonstrate that, after stimulation of secretion, intact as well as empty and partly empty secretory vesicles are present^7^. Quantitative TEM on stimulated and resting bovine chromaffin cells of the adrenal cortex showed no significant change in the number of peripheral dense-core vesicles after stimulation of secretion^2^. Similarly, combined studies using atomic force microscopy (AFM) and TEM clearly demonstrate no change in the total number of secretory vesicles following secretion in pancreatic acinar cells^10^. Fusion pores (porosomes) or depressions in pancreatic acinar- or GH-secreting cells are cone-shaped structures at the plasma membrane, with a 100- to 150-nm-diameter opening^3,6^. Membrane-bounded secretory vesicles ranging in diameter from 0.2 to 1.2 µm dock and fuse at depressions to release vesicular contents. Following fusion of secretory vesicles at depressions, a 20-40% increase in depression diameter has been demonstrated. It has therefore been concluded that secretory vesicles “transiently” dock and fuse at depressions^11^. In contrast to accepted belief, if secretory vesicles were completely incorporated at depressions, the fusion pore would distend much wider than what is observed. Additionally, if secretory vesicles were completely fused at the plasma membrane, there would be a decrease in total number of vesicles after secretion. Examination of secretory vesicles within cells before and after secretion in pancreatic acinar cells^10^ demonstrates that, although the total number of secretory vesicles remains unchanged after secretion, the number of empty and partly empty vesicles increases significantly, supporting the occurrence of transient fusion^10^. Earlier studies on mast cells also demonstrated an increase in the number of spent and partly spent vesicles following stimulation of secretion, without any demonstrable increase in cell size^1^. Other supporting evidence favoring transient fusion is the presence of neurotransmitter transporters at the synaptic vesicle membrane. These vesicle-associated transporters would be of little use if vesicles were to fuse completely at the plasma membrane to be endocytosed at a later time. Although the fusion of secretory vesicles at the cell plasma membrane occurs transiently, complete incorporation of membrane at the cell plasma membrane takes place when cells need to incorporate signaling molecules like receptors, second messengers and ion channels^11^. Therefore, in GH-secreting cells, transient fusion is suggested and no change in total number of vesicles is hypothesized. To test this hypothesis, the present study has been undertaken.

## MATERIALS AND METHODS

### Preparation of cell cultures

Newborn pigs, 1–8 days of age, were killed with electrical shock and decapitated. Pituitary glands were immediately removed^12^. The total number of animals used was 32. The pituitary glands were collected in cold sterile EBSS solution (4 °C). Anterior lobes were transferred to a sterile cold (4 °C) MEM–0·1% BSA medium. Primary cell cultures from neonatal anterior pituitary gland were established using a modified method of Huettner & Baughman^13^. Tissues from two animals were incubated for 50 min at 37 °C in 2 ml EBSS–papain solution (1 × 54 mg/ml). After incubation, the tissue was rinsed with EBSS solution and incubated for 5 min in trypsin-inhibitor solution (1 mg/ml). After being rinsed, once with EBSS solution and once with DMEM–0·1% BSA medium, the tissue was mechanically dispersed in DMEM–0·1% BSA medium by triturating through a fire-polished glass pipette. The undigested tissue was allowed to sediment. The supernatant containing cells was removed and filtered through a sterile filter. Cells were plated onto poly-L-lysine (0·1 mg/ml; 100 000 kDa)-coated glass coverslips (at a density of 2 × 10^5^ cells). Cells were allowed to attach to coverslips and, after 3-4 h, DMEM-0·1% BSA medium was exchanged with DMEM medium supplemented with 10% HS and 1 ml penicillin–streptomycin solution per 100 ml medium. Cultures were maintained at 37 °C in a humidified 5% CO_2_/95% air atmosphere. Experiments were carried out after 2 days in culture. The presence of somatotropes was confirmed by immunocytochemical methods.**

### Immunocytochemistry staining for L-692,585 or ghrelin-treated cells

After fixation with 4% paraformaldehyde for 30 min at room temperature, cells were incubated for 30 min in a 50% goat serum solution containing 1% BSA and 100 mM L-lysine to block non-specific binding and 0·4% Triton 626 X-100 to permeabilize the membrane. To stain the somatotropes, cultures of anterior pituitary gland were incubated in polyclonal anti-porcine GH antibody (dilution 1:50 000). Antibody visualization was accomplished by using a Vectastain ABC kit (Vector) and the nickel-enhanced 3,3΄ diaminobenzidine method^14^.

### Intracellular calcium imaging

The effect of secretagogues of L-692,585 or ghrelin, on intracellular calcium concentration [Ca^2+^]_i_ was evaluated by ratiometric imaging techniques^15^. Cells were loaded with Fura 2-AM for 40-60 min at 37 °C. One microliter of 25% (w/w) of Pluronic F-127 was mixed with 4 nM of AM ester to aid solubilization of the ester into aqueous medium. Coverslips containing pituitary cells were washed with HEPES-buffered solution and further incubated for 10 min at 37 °C to allow de-esterification of Fura 2-AM. All image processing and analyses were performed using an Attofluour system (Atto Bioscience, Rockville, MD, USA) with a Zeiss microscope. Background subtraction and ratio images were used to calculate the [Ca^2+^]_i _according to Equation 5 of Grynkiewitz et al^16^. Using wavelengths of 340 and 380 nm, Fura 2-AM was excited and the emitted light was collected at 520 nm.**

### Experimental animals

Neonatal Yorkshire pigs, 1-3 days of age, from the Iowa State University Animal Nutrition Farm, were euthanized and decapitated. Pituitary glands were immediately removed and cut sagittally in half. Animal care and experimental protocols were approved by IACUC.

### Tissue preparation

**Figure 1:**
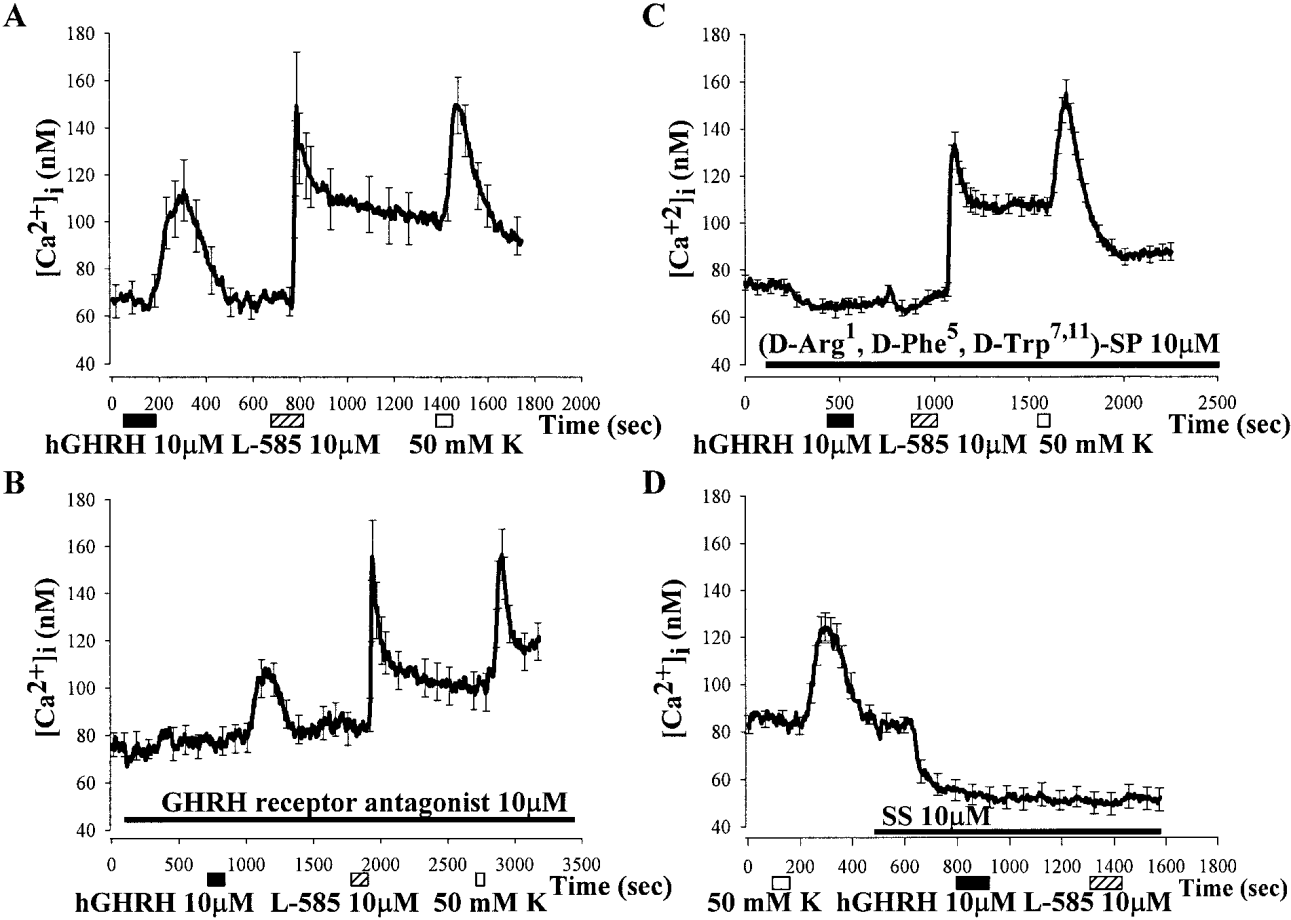
Intracellular calcium [Ca2+]i nM concentration after exposure to hGHRH to identify GH secreting cells, and followed by exposure to 10 mm L692,585(A). In the presence of GHRH receptor antagonist [Ca^2+^]_i_ nM concentration is greatly decreased whereas L692,585 calcium transit remains robust (B). Intracellular calcium [Ca^2+^]_i_ nM concentration after exposure to hGHRH to identify GH secreting cells, and followed by exposure to 10 mM L692,585. Substance P analogue [(D-Arg^1^,D-Phe^5^, D-Trp^7,11^]-SP-blocked the effect of GHRH, but not the stimulatory effect of L-692,585 on intracellular calcium response (C). Somatostatin (SS) completely blocked the effect of L692,585 on intracellular Ca^2+^ concentration. In the presence of a specific receptor antagonist to the GHS-R, the response of [Ca^2+^]_i_ to L692,585 was dampened (D).

The anterior lobe of the pituitary was cut into less than 1 mm cubes and each sagittal half of the gland was exposed to PBS or the GH secretagogue, L-692,585, 20 µM for 90 s. This is the same period of time in which L-692,585 evoked a marked increase in intracellular calcium concentration^12^ (**Figure 1**).

### TEM methods to optimize structure

The minced pituitary gland exposed to PBS or L-692,585 was fixed in 4% paraformaldehyde and 2.5% glutaraldehyde for 2 h and then transferred to 1% osmium tetroxide for 1 h. During the last step of graded acetone dehydration (25-100%), tissue was allowed to warm (24 C) for infiltration and embedding in an Epon-Araldite resin. Thin 40-70 nm sections were doubly stained with uranyl acetate and lead citrate.

### TEM methods to optimize antibody localization

The minced pituitary gland exposed to PBS or L-692,585 was fixed in 2% paraformaldehyde and 0.02% glutaraldehyde for 2 h and transferred to 0.15 м glycine for 1 h to bind free aldehyde groups. Then, tissue was dehydrated in graded methanol (25-90%, a drop at a time and temperature was lowered step-wise from 2 C to -20 C), infiltrated and embedded in Unicryl resin. Sections were cut at 40-70 nm.

### Immunogold labeling and staining

Sections were incubated on ovalbumin for 10 min, followed by overnight incubation at 2 C with anti-porcine GH antibody raised in rabbit. After washing in 0.01 M PBS, sections were incubated at room temperature on gold conjugated rabbit IgG raised in goat for 1 h. Sections were stained with 2% uranyl acetate for 15 min and then with lead citrate for 40 s. The optimal concentration for both antibodies was determined by systematically varying the concentration until minimal background was obtained. Controls, PBS substituted for the primary antibody, and incubation with only the gold (10 nm in diameter) conjugated second antibody, showed minimal background labeling. Thin sections were examined at 80 kV in a JEOL JEM-100CXII electron microscope. Images were recorded on Kodak SO-163 electron image film.

### Quantitative analysis 

Negatives were scanned and the total number of filled, empty and partly empty vesicles was counted independently in the prints by two persons. The number of vesicles in each of the three categories is listed per µm^2^ of cell area.

### Statistical analysis

Means of the number of vesicles in each category per µm2 were obtained for PBS- and L-692,585-treated groups. Data are expressed as the mean ± SEM. All data were subjected to ANOVA to establish whether significant differences (P < 0.001 or P < 0.05) were present, in which case P values for pair-wise differences between groups were calculated by using the Student’s t test.

## RESULTS

### Stimulatory Effect of L-692,585 or Ghrelin on [Ca^2+^]_i_ in Cultured Porcine Somatotropes

The presence of somatotropes in pituitary cell cultures was confirmed by immunocytochemical staining with an antibody raised against GH. GH-immunoreactive cells comprised 40% of the total pituitary cells in cultures. An increase in [Ca^2+^]_i_ above baseline following hGHRH application functionally identified GH cells. Perfusion with hGHRH (10 µM) for 2 min increased the [Ca^2+^]_i_ in somatotropes by 55 ± 3.0 nM (n = 232; p < 0.01; **Figure 1** [A]). A GHRH receptor antagonist did not inhibit the stimulatory effect of L-692,585 (**Figure 1** [B]). Of the cells that responded to hGHRH, 98% also responded to 1 µM ghrelin applied 10 min after the application of hGHRH. Perfusion application of 1 µM ghrelin for 2 min produced a prompt transient increase in [Ca^2+^]_i_ of 57 ± 3.0 nM (n = 227; p < 0.01), followed by a sustained decline to a plateau above the basal level.

### Receptor Mediation of the Stimulatory Effect of Ghrelin on [Ca^2+^]_i_

To determine whether the effect of ghrelin was receptor mediated, experiments were performed with (D-Lys3)-GHRP-6, a specific antagonist of GHS-R. Perfusion of cultures with 100 µM (D-Lys3)-GHRP-6 for 10 min before ghrelin decreased the [Ca^2+^]_i_ from 91 ± 4.8 to 64 ± 2.8 nM (n = 101; p < 0.01). In the presence of (D-Lys3)-GHRP-6, the increase in [Ca^2+^]_i_ evoked by ghrelin (1 µM) was delayed for about 100 s and decreased as compared with the response in controls (81 ± 4.7 nM, n = 92, vs. 65 ± 4.0 nM, n = 80; p < 0.05). In control cultures, 98% of the cells (92 of 94) that responded to hGHRH responded to ghrelin, while after pretreatment of cultures with (D-Lys3)-GHRP-6, only 79% of the cells (80 of 101) that responded to hGHRH responded also to ghrelin. These results support the GHS-R, mediating the effect of ghrelin increasing the [Ca^2+^]_i_ in cultured porcine somatotropes.

**Figure 2:**
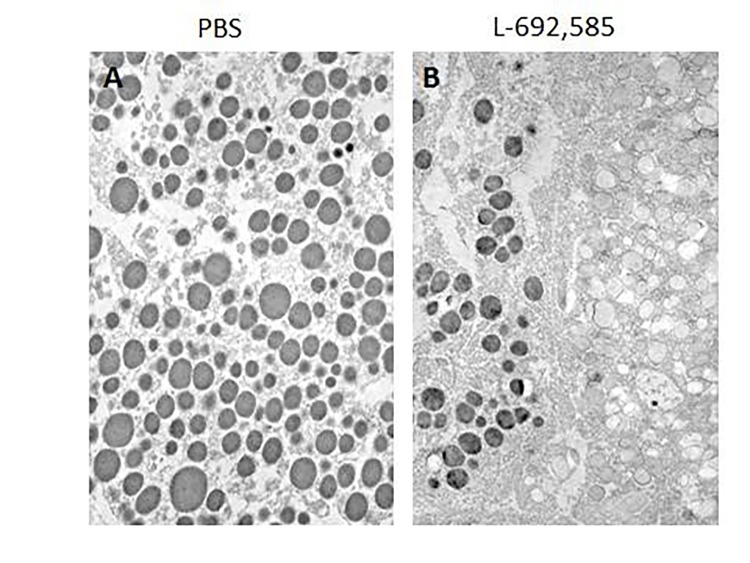
TEM images of immunogold labeled secretory vesicles in GH cells Note localization of GH is only in electron dense vesicles in both control (resting) (A) and stimulated (B) cells. After stimulation of GH secretion with the secretagogue, L-692,585, for 90 s, there was complete absence of immunogold labeled GH-antibody in empty vesicles (B), Magnification, × 28,000.

Control (resting) pituitary cells exposed to PBS contained more than twice as many filled vesicles than did the stimulated cells exposed to the GH secretagogue, L-692,585 (4.9 ± 0.21 in control, 2.3 ± 0.23 in stimulated; P < 0.001). Stimulated cells contained nearly twice as many empty vesicles (0.6 ± 0.13 in control, 1.2 ± 0.16 in stimulated; P < 0.05) and 2.5 times more partly empty vesicles than did control cells (1.1 ± 0.08 in control, 2.6 ± 0.12 in stimulated; P < 0.001). There was no significant difference in total number of vesicles between control and stimulated pituitary cells. The remarkable increase in number of empty and partly empty vesicles in stimulated cells compared with control cells is evident in **Figure 2** [A,B]. Immunogold labeling with GH-antibody occurs in only electron dense GH vesicles in both control and stimulated cells (**Figure 2** [A,B]). There was an absence of immunogold labeling with GH-antibody in empty vesicles or other areas of the cytoplasm. The results of stimulating live GH cells for 90 s with L-692,585 is that filled secretory vesicles empty very rapidly, most likely by docking at the plasma membrane, releasing vesicular contents, and then the empty or partly empty vesicles return to the cytoplasmic compartments of the cell.

## DISCUSSION

The final step of exocytosis has been reconsidered against the commonly accepted process, “total fusion” involving total incorporation of the secretory vesicle membrane into the cell plasma membrane and the compensatory retrieval of excess membrane by endocytosis at a later time. The majority of the electrophysiological measurements, together with current TEM and AFM studies demonstrates that membrane bounded secretory vesicles transiently dock and fuse at the fusion pore (porosome) to release their contents. A step increase in membrane capacitance also may be due to secretory vesicles undergoing “transient fusion” at the plasma membrane during secretion. In slow secreting cells, (*i.e*., pancreatic acinar cell in rats) membrane capacitance involves only transient fusion events after stimulation of secretion. In fast secreting cells such as nerve or mast cells, the number of secretory vesicles fusing at the plasma membrane at one time is likely much greater than in pancreatic acinar cells. The sequential fusion of secretory vesicles before their dissociation from the plasma membrane likely encounters a step increase in plasma membrane capacitance in nerve cells that also require a rapid and selective retrieval of vesicle membrane in addition to rapid fusion of synaptic vesicles at the presynaptic membrane. Both rapidity and high energy are required; exocytosis in neurons or neuroendocrine cells likely depends on transient fusion of vesicles at the membrane fusion pore (porosome). Continuous exocytosis followed by membrane retrieval would permit endocrine cells to efficiently maintain secretory activity for long periods of time, thus maintaining a constant cell membrane area. Rat pituitary GH cells undergo continuous exocytosis and membrane retrieval that persists in whole-cell recording^17^. Majó and colleagues^18^ suggested similar secretory mechanisms for synaptic vesicles and secretory organelles in both neuronal and endocrine cells that have a highly regulated secretory pathway for intracellular communication. The secretory vesicles that fuse with the plasma membrane in response to a physiological stimulus show similarities of synaptic proteins in both the anterior pituitary and the nerve terminal. Although several secretory vesicle-associated proteins have been implicated in exocytosis, none is incorporated into the plasma membrane as would occur if total fusion occurred^5,8^. From our study on GH cells, 90 s exposure to 20 µМ L-692,585 (GH-secretagogue) for stimulation of GH secretion was ideal in inducing rapid and effective exocytosis. L-692,585 intravenously administered to pigs causes an immediate peak release of GH > 80 ng/ml peripheral plasma within 5 min compared with normal pulsatile GH peaks of 6-10 ng/ml in placebo treated controls^19^. Intracellular signal transduction of GH-secretagogue undergoes a phosphoinositol-protein kinase C pathway that induces intracellular Ca^2+^ accumulation and depolarization, leading to exocytosis of GH-containing vesicles^12^.

Ghrelin is an endogenous ligand for GH secretagogue receptor (GHS-R) and is predominantly produced by the stomach and lower amounts in the hypothalamus and various peripheral tissues. Ghrelin is a potent stimulator of GH secretion from the pituitary in vivo and in vitro. GH secretion from the pituitary also is controlled by two hypothalamic peptides: stimulatory GH-releasing hormone (GHRH) and inhibitory somatostatin-14 (SRIH). GH participates in its own rhythmic secretion through feedback action on GHRH and SRIH neurons. The ability of ghrelin to induce an increase in the intracellular Ca^2+^ concentration – [Ca^2+^]_i_ – somatotropes was examined in dispersed porcine pituitary cells using a calcium imaging system^20^ (**Figure 1**). Somatotropes were functionally identified by application of human GH-releasing hormone (hGHRH). Ghrelin increased the [Ca^2+^]_i_ in a dose-dependent manner in 98% of the cells that responded. In the presence of (D-Lys3)-GHRP-6, a specific receptor antagonist of GHS-R, the increase in [Ca^2+^]_i_ evoked by ghrelin was decreased. Pretreatment of cultures with somatostatin or neuropeptide Y reduced the ghrelin-induced increase of [Ca^2+^]_i_. The stimulatory effect of ghrelin on somatotropes was greatly attenuated in low calcium saline and blocked by nifedipine, an L-type calcium channel blocker, suggesting involvement of calcium channels. In a zero Na+ solution, the stimulatory effect of ghrelin on somatotropes was decreased, suggesting that besides calcium channels, sodium channels are also involved in ghrelin-induced calcium transients. Either SQ-22536, an adenylyl cyclase inhibitor, or U73122, a phospholipase C inhibitor, decreased the stimulatory effects of ghrelin on [Ca^2+^]_i_ transiently, indicating the involvement of adenylyl cyclase-cyclic adenosine monophosphate and phospholipase C inositol 1,4,5-trisphosphate pathways. The non-peptidyl GHS, L-692,585, induced changes in [Ca^2+^]_i_ similar to those observed with ghrelin. Application of L-692,585 after ghrelin did not have additive effects on [Ca^2+^]_i_. Preapplication of L-692,585 blocked the stimulatory effect of ghrelin on somatotropes. Our results suggest that the actions of ghrelin and synthetic GHS closely parallel each other, in a manner that is consistent with an increase of hormone secretion. An understanding of the molecular mechanisms by which ghrelin and GHS modulate GH secretion is of particular interest in the regulation of GH for muscle accretion and somatic growth.

The step increase in plasma membrane capacitance of GH cells, therefore, may result from a rapid transient fusion which is consistent with the AFM observation that shows the presence of ‘pits’ which contain ‘depressions’ or fusion pores at the GH cell plasma membrane that increase markedly in diameter after L-692,585 exposure. The data reported herein clearly show that the total number of secretory vesicles in porcine pituitary cells is not decreased after exocytosis and is consistent with a mechanism that vesicles transiently dock and fuse at the fusion pore to release vesicular contents after stimulation of secretion. Three categories of vesicles: filled, empty and partly empty were identified. Resting GH cells contain more than twice as many filled vesicles than did the stimulated ones. Stimulated cells, however, contained nearly as many empty vesicles and 2.5 times more partly empty vesicles than did resting cells. The total number of secretory vesicles did not change after secretion.

Transport of vesicles to the plasma membrane is powered by high-energy molecules such as ATP, GTP or NADH. Thus, transport of a GH secretory vesicle is along microtubules, which is driven by kinesin motor proteins and fueled by ATP^21,22^. The speed of kinesin on a microtubule is about 3 mm s^-1^ and a single step is roughly 8 nm, therefore, this motor protein makes about 375 steps per second, with each step requiring the consumption of one ATP molecule^21-23^. Given that ~1000 secretory vesicles are transported on microtubules of a GH cell at every instant of time^17,18^, the total rate of energy consumption as a result of vesicle transport is approximately 3.75 × 10^5^ ATP molecules per second^21-23^. Since the total number of ATP molecules in a cell at any given moment is ~109, and since these molecules are used up and completely replaced in ~1-2 min, the total rate of consumption by the GH cells is approximately 107 ATP molecules per second^21-23^. Thus, the kinesin-on-microtubule motors use ~4% of the cell’s ATP fuel by transporting 1000 vesicles. This is a modest estimation of the number of secretory vesicles in a GH cell that secretes on stimulation. These results from TEM are consistent with a mechanism that, after stimulation of secretion, vesicles transiently dock and fuse at the fusion pore (porosome) to release vesicular contents. The molecular mechanism of secretory vesicle fusion at the base of porosomes, and the regulated expulsion of intravesicular contents during cell secretions, are also resolved. Based on these and other supporting findings, transient fusion of secretory vesicles at the fusion pore (porosome) on the plasma membrane may be universal in the process of exocytosis.

In our laboratory, studies on growth hormone (GH) cells of the pig pituitary gland using a combination of AFM, EM, immunochemical and fluorometric analysis demonstrate the presence of porosomes at the cell plasma membrane, where secretory vesicles transiently dock and fuse to expel growth hormone to the outside^6,24,25^. Porosomes in resting GH cells measure 154 ± 4.5 nm (mean ± SE) in diameter and, following stimulation of secretion, increase their diameter by 40% (215 ± 4.6 nm; P < 0.01)^6^. The enlargement of porosome diameter during cell secretion and its subsequent decrease, accompanied by the loss in growth hormone secretion, implied that they constituted the secretory machinery in the GH cells of the pituitary. A direct determination that porosomes are the secretory portal in GH cells, by which growth hormone is expelled, was unequivocally demonstrated using immuno-AFM studies, where gold-tagged growth hormone-specific antibody was found to selectively localize at the porosome openings following stimulation of secretion^6^. These studies established porosomes as the secretory portals in the GH cells of the pituitary gland. Our EM studies further demonstrated that, following GH cell stimulation, the number of filled vesicles decreases and there is a large increase in the number of partially empty secretory vesicles. However, there is no appreciable change in the total number of secretory vesicles following GH secretion^6^. These results further attest to the fact that secretory vesicles transiently dock and fuse at the porosome base in GH cells of the pig pituitary gland in the process of growth hormone release, a paradigm shift in our understanding of growth hormone secretion, and secretion in general.

In conclusion, one needs to recognize that, just in the near past, our understanding of the breakdown of waste products in cells was centered on the lysosome. Now we have learned of the existence of another type of garbage disposal system, called the proteasome, which degrades misfolded or damaged proteins in cells. Similarly, the discovery of a new cellular structure, the porosome, at the cell plasma membrane has helped redefine the process of secretion in cells. The porosome structure has, furthermore, provided the molecular underpinnings for the kiss-and-run form of release of the secretory products of cells.

- **Stimulation of live GH cells for 90s with L-692,585 leads to the rapid emptying of the filled secretory vesicles, by docking at the plasma membrane, releasing vesicular contents. Then the empty or partly empty vesicles most likely return to the cytoplasmic compartments of the cell.**
- **No change in total number of secretory vesicles occurs following secretion**
